# Erring Characteristics of Deformable Image Registration-Based Auto-Propagation for Internal Target Volume in Radiotherapy of Locally Advanced Non-Small Cell Lung Cancer

**DOI:** 10.3389/fonc.2022.929727

**Published:** 2022-07-22

**Authors:** Benjamin J. Rich, Benjamin O. Spieler, Yidong Yang, Lori Young, William Amestoy, Maria Monterroso, Lora Wang, Alan Dal Pra, Fei Yang

**Affiliations:** ^1^ Department of Radiation Oncology, University of Miami, Miami, FL, United States; ^2^ Department of Radiation Oncology, The First Affiliated Hospital of University of Science and Technology of China, Hefei, China; ^3^ Department of Radiation Oncology, University of Washington, Seattle, WA, United States

**Keywords:** ITV generation, auto-propagation, deformable propagation, 4DCT, LA-NSCLC

## Abstract

**Purpose:**

Respiratory motion of locally advanced non-small cell lung cancer (LA-NSCLC) adds to the challenge of targeting the disease with radiotherapy (RT). One technique used frequently to alleviate this challenge is an internal gross tumor volume (IGTV) generated from manual contours on a single respiratory phase of the 4DCT *via* the aid of deformable image registration (DIR)-based auto-propagation. Through assessing the accuracy of DIR-based auto-propagation for generating IGTVs, this study aimed to identify erring characteristics associated with the process to enhance RT targeting in LA-NSCLC.

**Methods:**

4DCTs of 19 patients with LA-NSCLC were acquired using retrospective gating with 10 respiratory phases (RPs). Ground-truth IGTVs (GT-IGTVs) were obtained through manual segmentation and union of gross tumor volumes (GTVs) in all 10 phases. IGTV auto-propagation was carried out using two distinct DIR algorithms for the manually contoured GTV from each of the 10 phases, resulting in 10 separate IGTVs for each patient per each algorithm. Differences between the auto-propagated IGTVs (AP-IGTVs) and their corresponding GT-IGTVs were assessed using Dice coefficient (DICE), maximum symmetric surface distance (MSSD), average symmetric surface distance (ASSD), and percent volume difference (PVD) and further examined in relation to anatomical tumor location, RP, and deformation index (DI) that measures the degree of deformation during auto-propagation. Furthermore, dosimetric implications due to the analyzed differences between the AP-IGTVs and GT-IGTVs were assessed.

**Results:**

Findings were largely consistent between the two algorithms: DICE, MSSD, ASSD, and PVD showed no significant differences between the 10 RPs used for propagation (Kruskal–Wallis test, *ps* > 0.90); MSSD and ASSD differed significantly by tumor location in the central–peripheral and superior–inferior dimensions (*ps* < 0.0001) while only in the central–peripheral dimension for PVD (*p* < 0.001); DICE, MSSD, and ASSD significantly correlated with the DI (Spearman’s rank correlation test, *ps* < 0.0001). Dosimetric assessment demonstrated that 79% of the radiotherapy plans created by targeting planning target volumes (PTVs) derived from the AP-IGTVs failed prescription constraints for their corresponding ground-truth PTVs.

**Conclusion:**

In LA-NSCLC, errors in DIR-based IGTV propagation present to varying degrees and manifest dependences on DI and anatomical tumor location, indicating the need for personalized consideration in designing RT internal target volume.

## Introduction

Non-small cell lung cancer (NSCLC) presents with locally advanced disease in approximately one-third of cases (1). For patients with adequate performance status, standard-of-care treatment for unresectable locally advanced NSCLC (LA-NSCLC) begins with concurrent chemoradiation therapy, but outcomes remain poor (2, 3). In addition, definitive radiotherapy (RT) for LA-NSCLC has a high rate of toxicity, particularly cardiac, limiting survival outcomes (3). Therefore, to optimize outcomes for LA-NSCLC, it is critical for technological advances to improve the delivery of definitive RT with the intention of increasing disease control while limiting toxicity (4).

The respiratory motion of lung tumors adds to the challenge of targeting disease with RT while minimizing the dose to the nearby organs at risk (OARs) (5). Contouring a gross tumor volume (GTV) and clinical target volume (CTV) on a free-breathing computed tomography (CT) scan with a universally expanded planning target volume (PTV) can result in radiation geographic miss and volume distortion due to respiratory motion (6). A universal PTV expansion also ignores nuances in demographic and disease characteristics that influence respiratory tumor motion (7, 8). Multiple methods are available to account for lung tumor respiratory motion during RT (9). These methods include deep inspiratory breath-hold, spontaneous respiratory gating, and the creation of a personalized internal target volume (ITV). Deep inspiratory breath-hold and spontaneous respiratory gating increase treatment time and require patient compliance, which can be burdensome for patients receiving 6 weeks of RT for LA-NSCLC (10). In contrast, treatment with an ITV that accounts for intrathoracic target motion allows the patient to be treated while free-breathing. Constructing an ITV has dosimetric implications that can improve the therapeutic ratio, particularly with more conformal RT techniques such as intensity-modulated RT (IMRT) (11).

To generate an ITV, lung tumor motion is characterized with a four-dimensional CT (4DCT) scan. The 4DCT is based on three-dimensional CT (3DCT) images correlated with the breathing cycle and a sorting algorithm that reconstructs the 4DCT by dividing the 3DCT images into a fixed number of respiratory phase (RP)-based bins (12). In one method, a target is contoured on each 3DCT RP image, and their union on the 4DCT creates the ITV (13). In this manner, an internal GTV (IGTV) is created through the fusion of contoured GTVs across the RP 3DCT images. However, manually contouring the GTV on each RP 3DCT image to create an IGTV adds pressure on already strained clinical resources, rendering it impractical, if not impossible, for routine practice. In addition, variability in manual contour delineation between phases can introduce uncertainty in the treatment planning margins (14, 15). Auto-propagation of GTV contours between phases has been proposed as an efficient solution to getting around manually contouring the GTV on each RP (16, 17). The current available auto-propagation algorithms rely on either deformable models (18) or deformable image registration (DIR) models (19). DIR is a technique that elastically deforms images to generate a map of corresponding features, which allows for tracking of intrathoracic tumor motion during respiration. Prior studies evaluating the accuracy of auto-propagated IGTVs compare them with an expert physician’s manually contoured IGTVs, or the ground-truth IGTV (GT-IGTV) (16, 17, 20, 21). For example, Speight et al. demonstrated that IGTVs generated using commercially available auto-propagation DIR segmentation were accurate compared to GT-IGTVs (20). However, these studies are limited to early-stage NSCLC and did not explore the impact of anatomical tumor location or the RP used to initialize auto-propagation on the accuracy of the generated IGTVs.

To our knowledge, little research has been done to assess erring patterns associated with DIR-based IGTV auto-propagation in LA-NSCLC. Given the paucity of data, this study aimed to evaluate the accuracy of commercially available commonly accepted DIR-based auto-propagation algorithms for generating IGTVs in LA-NSCLC for to identify erring characteristics associated with the process that might have implications for RT targeting.

## Materials and Methods

### Imaging Cohort

The study cohort consisted of patients accrued within the 4D-lung data collection for which imaging is publicly available from The Cancer Imaging Archive (TCIA) (22). The local institutional review board (IRB) waived this study from review on account of only publicly available aggregated patient data being utilized. The dataset comprised 4D fan-beam CT (4D-FBCT) of 19 LA-NSCLC patients undergoing concurrent chemoradiotherapy at the Virginia Commonwealth University Massey Cancer Center (Richmond, VA), from 2008 through 2012 (23, 24). 4D-FBCT images were acquired on a 16-slice helical CT simulator (Brilliance Big Bore, Philips Medical Systems, Andover, MA) as respiration-correlated CTs during simulation, prior to therapy. The respiratory signal was obtained with the aid of an external respiratory motion surrogate (Real-time Position Management, Varian Medical Systems, Palo Alto, CA), and audiovisual feedback was performed during all 4D-FBCT acquisitions. The acquired raw data were sorted according to the respiratory phase into 10 bins, with each constituting a separate 3DCT dataset. These bins represented 10 equally spaced points in time across the full respiratory cycle and were linearly defined in increments of 10%, with 0% representing peak inhalation and 50% end of exhalation for all patients. CT exposures were performed at 120 kVp with modulated mAs (50 to 114 mA; 3.53 to 5.83 ms). The final reconstructed scans featured a slice thickness of 3 mm and in-plane spacing from 0.98 to 1.17 mm. In addition, an average intensity projection (AIP) scan was derived from the 4DCT for each of the studied patients.

### Segmentation of Ground-Truth IGTVs

The dataset included GTVs already delineated on each of the 10 respiratory phases for all 19 patients by radiation oncologists with expertise in treating thoracic malignancies. To repurpose the accompanying contouring data for ITV propagation, as was of interest in the current study, the GTV contours were further reexamined and revised, if necessary, according to the most updated guidelines (25) by a radiation oncologist with extensive expertise in contouring lung lesions as part of RT planning. The primary tumor was assessed under standard lung window/level (−600/1,600 HU) for borders adjacent to the lung and under mediastinal window/level (+20/400 HU) for borders adjacent to the mediastinum. Lymph nodes were also inspected under mediastinal window/level for involvement. The estimated ground-truth GTV (GT-GTV) on one single RP for a given patient was created by combining the primary tumor mass volume and the involved nodal disease volume as defined on the corresponding 3DCT. This was carried out to generate GT-GTVs on all 10 respiratory phases for each of the 19 patients. For each patient, the union of GT-GTVs across all 10 respiratory phases was defined as the ground-truth IGTV (GT-IGTV).

### IGTV Auto-Propagation and Evaluation

The software platforms used for IGTV auto-propagation included MIM Maestro^®^ v7.1.3 (MIM Software, Cleveland, OH) and Velocity^™^ oncology imaging informatics system v4.1 (Varian Medical Systems, Palo Alto, CA). For every patient, DIR-based IGTV propagation was carried out for each of the GT-GTVs from all 10 phases, resulting in 10 separate auto-propagated IGTVs (AP-IGTVs) for each software platform. As of the entire cohort, there were 190 AP-IGTVs for each software and 380 AP-IGTVs in total. The DIR algorithm adopted in MIM Maestro^®^ for IGTV auto-propagation is the VoxAlign Deformation Engine^®^, and it employs a coarse-to-fine multi-resolution approach in the search for the best corresponding locations in the target image for the predefined control points on the source image (26). The image matching metric it uses to minimize intensity differences between the two images is optimized based on a gradient descent method regularized to avoid tears and folds in the deformation field. On the other hand, for Velocity^™^, it recourses to the multi-resolution elastic B-spline deformation algorithm for IGTV auto-propagation (27). It matters mutual information as the cost function metric in estimating the marginal and joint probability density functions of the volumes across a subset of voxels, which then allows the optimizer to identify proper deformation. To generate each AP-IGTV, a single phase-binned 3DCT dataset containing a GT-GTV was fed as a reference to the deformation engines, which then sequentially propagated that GTV volume to the 3DCTs of the remaining nine respiratory phases. Finally, the obtained auto-propagated GTVs were combined with the reference GTV to form the AP-IGTV for the given reference phase. The accuracy of the AP-IGTVs relative to their respective GT-IGTV was assessed by aid of four commonly used volumetric and distance-based similarity metrics. These included (28–31):

*Dice coefficient*: measures the extent of spatial overlap between two volumes *V*_1_ and *V*_2_ such as:


$$DICE=2\times \frac{V_{1}\cap V_{2}}{\left|V_{1}\right|+\left|V_{2}\right|}.$$

*Maximum symmetric surface distance*: known also as the Hausdorff distance (32), estimates the maximum extent to which the surfaces of *V*_1_ and *V*_2_ differ and is defined as:


$$MSSD=max\left(max_{i=1}^{n_{SV1}}|d_{i}^{SV_{1},SV_{2}}|,max_{j=1}^{n_{SV2}}|d_{j}^{SV_{2},SV_{1}}|\right),$$

where *n_sv_*_1_ and *n_sv_*_2_ are the number of surface voxels on *V*_1_ and *V*_2_, $d_{i}^{SV_{1},SV_{2}}$ is the distance to the closest surface voxel of *V*_2_ for the *i*th surface voxel of *V*_1_, and $d_{j}^{SV_{2},SV_{1}}$ is the distance to the closest surface voxel of *V*_1_ for the *j*th surface voxel of *V*_2_.

*Average symmetric surface distance*: estimates the average extent to which the surfaces of *V*_1_ and *V*_2_ differ and is defined as:


$$ASSD=\frac{1}{n_{SV_{1}}+n_{SV_{2}}}\left(\sum_{i=1}^{n_{SV_{1}}}\left|d_{i}^{SV_{1},SV_{2}}\right|+\sum_{j=1}^{n_{SV2}}\left|d_{i}^{SV_{2},SV_{1}}\right|\right).$$

*Percent volume difference:* quantifies the percent difference between *V*_1_ and *V*_2_ with the latter being designates as the reference:


$$PVD=\frac{V_{1}-V_{2}}{V_{2}}\times 100.$$

Among these measures, DICE has values ranging from 0 to 1, where 0 indicates that the two volumes do not overlap while 1 indicates that they are spatially identical. MSSD and ASSD measure the maximum and average differences in millimeters between the surface of the two volumes, respectively, with the ideal value being 0 for both. Positive PVD values indicate that the obtained volume is larger than the reference volume and vice versa for negative values. In addition, a deformation index was defined and calculated for each auto-propagation as the volume ratio between AP-IGTV and the corresponding GT-GTV used to initialize auto-propagation such as:


$$DI=\frac{V_{AP–IGTV}}{V_{GT–GTV}}.$$

DI characterizes the extent of deformation, with values greater than, equal to, or less than 1 corresponding, respectively, to volume expansion, no volume change, and volume reduction.

### IGTV Spatial Allocation

IGTVs were allocated according to the anatomical location of the main tumoral mass. Central IGTVs were defined as those primary tumors within 2 cm of the proximal bronchial tree, major vessels (aorta, upper mediastinal vessels, and pulmonary artery extending to the tertiary bronchus), esophagus, heart, trachea, pericardium, brachial plexus, or vertebral body (33). IGTVs not meeting the criteria as a central lesion were defined as peripheral. In the craniocaudal dimension, IGTVs were divided into superior and inferior. Superior primary tumors were above the oblique and horizontal fissure (right and left upper lobes) while inferior primary tumors were inferior to the oblique and horizontal fissure (left and right lower lobes, right middle lobe).

### IGTV Dosimetric Characteristics

To characterize the dosimetric implications of differences in AP-IGTVs from the GT-IGTVs, a dosimetric analysis was performed. RT plans were created in Eclipse treatment planning software v16.1 (Varian Medical Systems, Palo Alto, CA) using the contouring guidelines and dose constraints in the RTOG 0617 protocol (3). For each patient, the AP-IGTV used for planning was the one that had DI closest to the overall median DI among all the AP-IGTVs created by both algorithms. Two CTVs were created from the GT-IGTV and AP-IGTV respectively using a 5-mm isotropic expansion cropped from organs at risk (OARs) and bone for each patient. PTVs were created using a 5-mm isotropic expansion from the corresponding CTVs. As a result, each patient had an auto-propagated PTV (AP-PTV) and ground-truth PTV (GT-PTV). A volumetric modulated arc therapy (VMAT) plan was created for each patient with prescription dose of 60 Gy in 30 fractions. The prescription had 100% of dose to 95% of the AP-PTV (V_100%_ ≥ 95%) and maximum dose less than 120%. The following OAR constraints were used: spinal cord maximum dose of 50.5 Gy, total lung volume receiving 20 Gy (V_20Gy_) less than 37% and mean dose less than 20 Gy, esophagus mean dose less than 34 Gy, heart volume receiving 60 Gy (V_60Gy_) less than 33%, volume receiving 45 Gy (V_45Gy_) less than 66%, and volume receiving 40 Gy (V_40Gy_) less than 100%. Plans were normalized so that 95% of the AP-PTV received 60Gy (i.e., 95% of prescription volume AP-PTV receiving 100% of prescribed dose of 60 Gy). The plans were reviewed to ensure meeting constraints. Dosimetric analysis of VMAT plans was performed on the GT-PTV for each patient, and dose volume histograms (DVHs) were generated. Specifically, V_100%_ was assessed of the GT-PTV with a value ≥ 95% considered sufficient for meeting the prescription constraint.

### Statistical Analysis

Continuous demographic characteristic variables of the studied cohort were described with mean and range, while the number and percentage of occurrences were used to describe the categorical variables. Statistical analysis was performed using JMP Pro^®^ Version 16 (SAS Institute Inc., Cary, NC) statistical software package. DICE, MSSD, ASSD, and PVD of the AP-IGTVs relative to their corresponding GT-IGTVs were examined to see if they differed between respiratory phases and between anatomical locations by the Kruskal–Wallis H omnibus test with the null hypothesis being that they remained invariant spatially and temporally (34). For significant results, pairwise comparisons were performed using the *post hoc* Dunn’s test with Bonferroni adjustment for multiple comparisons (35, 36). In addition, DICE, MSSD, ASSD, and PVD of the AP-IGTVs relative to their corresponding GT-IGTVs were examined for correlation with DI to investigate if the accuracy of auto-propagated IGTVs had any dependency on the extent of deformation through the use of Spearman’s rank correlation coefficient (R) (37). *P* values were determined for the calculated correlation coefficients to assess statistical significance, with the null hypothesis being that correlation coefficients did not differ significantly from zero. All statistical tests were two-sided, and *p* values of 0.05 or less, after multiple test corrections using the Bonferroni method if needed, were considered statistically significant.

## Results

**Figure 1:**
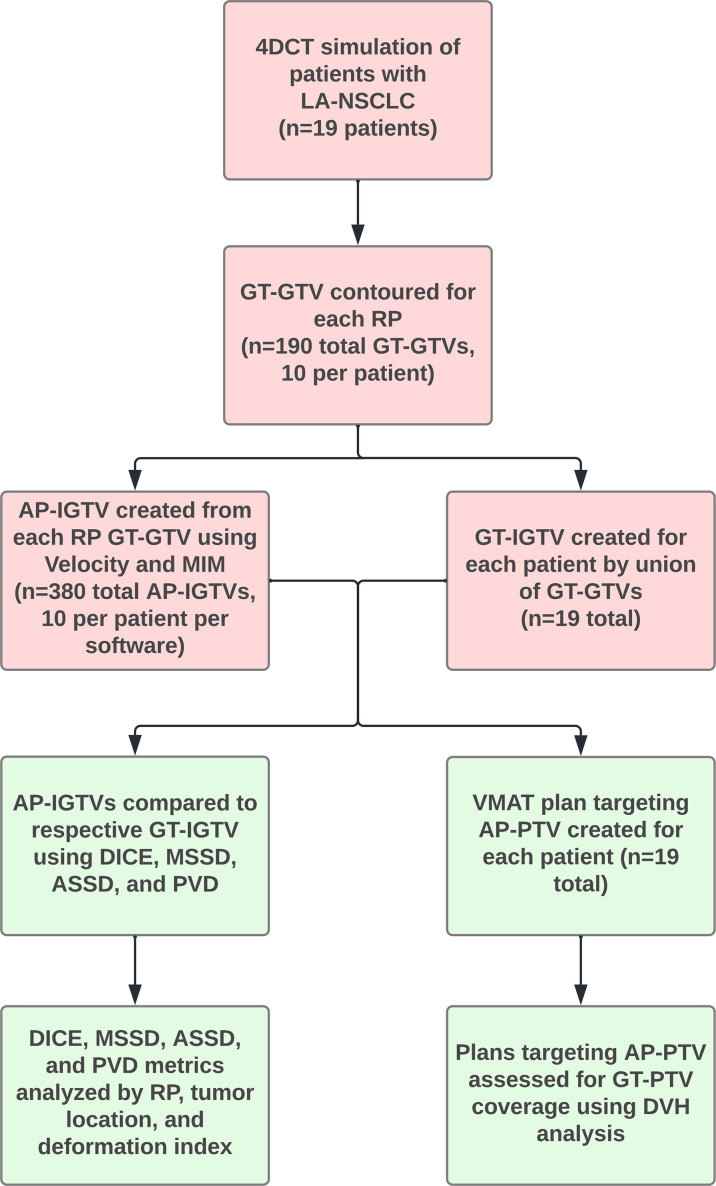
Flowchart with overview of study procedures. Abbreviations: 4D computed tomography (4DCT), auto-propagated internal gross tumor volume (AP-IGTV), auto-propagated planning target volume (AP-PTV), average symmetric surface distance (ASSD), Dice coefficient (DICE), dose volume histogram (DVH), ground-truth gross tumor volume (GT-GTV), ground-truth internal gross tumor volume (GT-IGTV), ground-truth planning target volume (GT-PTV), locally advanced non-small cell lung cancer (LA-NSCLC), maximum symmetric surface distance (MSSD), percent volume difference (PVD), respiratory phase (RP), and volumetric modulated arc therapy (VMAT).

**Figure 2:**
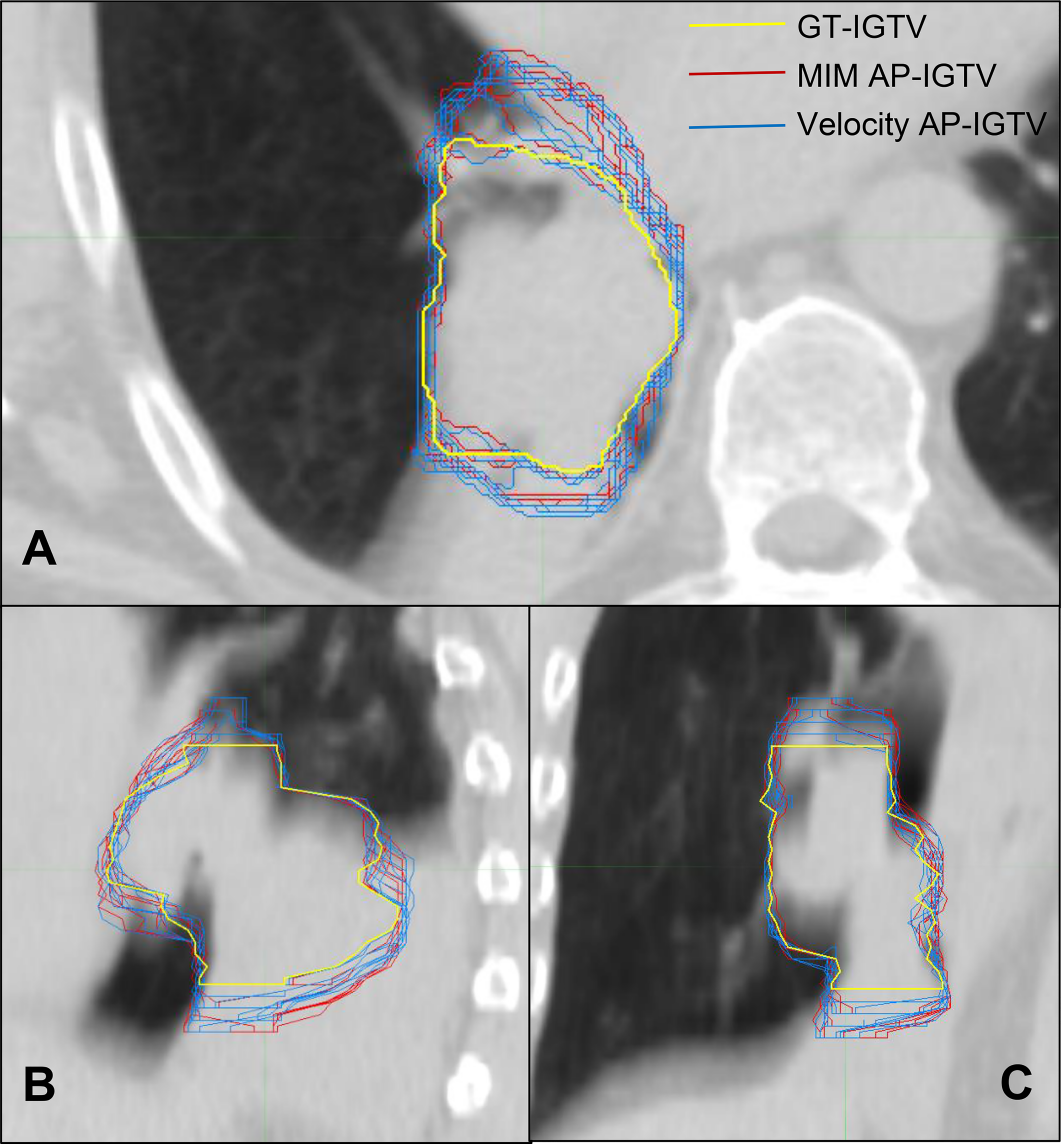
CT cross sections of one locally advanced non-small cell lung cancer (LA-NSCLC) lesion representative of the study cohort in axial **(A)**, sagittal **(B)**, and coronal **(C)** planes with variation in auto-propagated internal gross tumor volumes (AP-IGTV) derived from the two employed deformable image registration (DIR) algorithms—MIM (outlined in red) and Velocity (outlined in blue)—compared to the ground-truth internal gross tumor volume (GT-IGTV; outlined in thick yellow).

**Figure 3:**
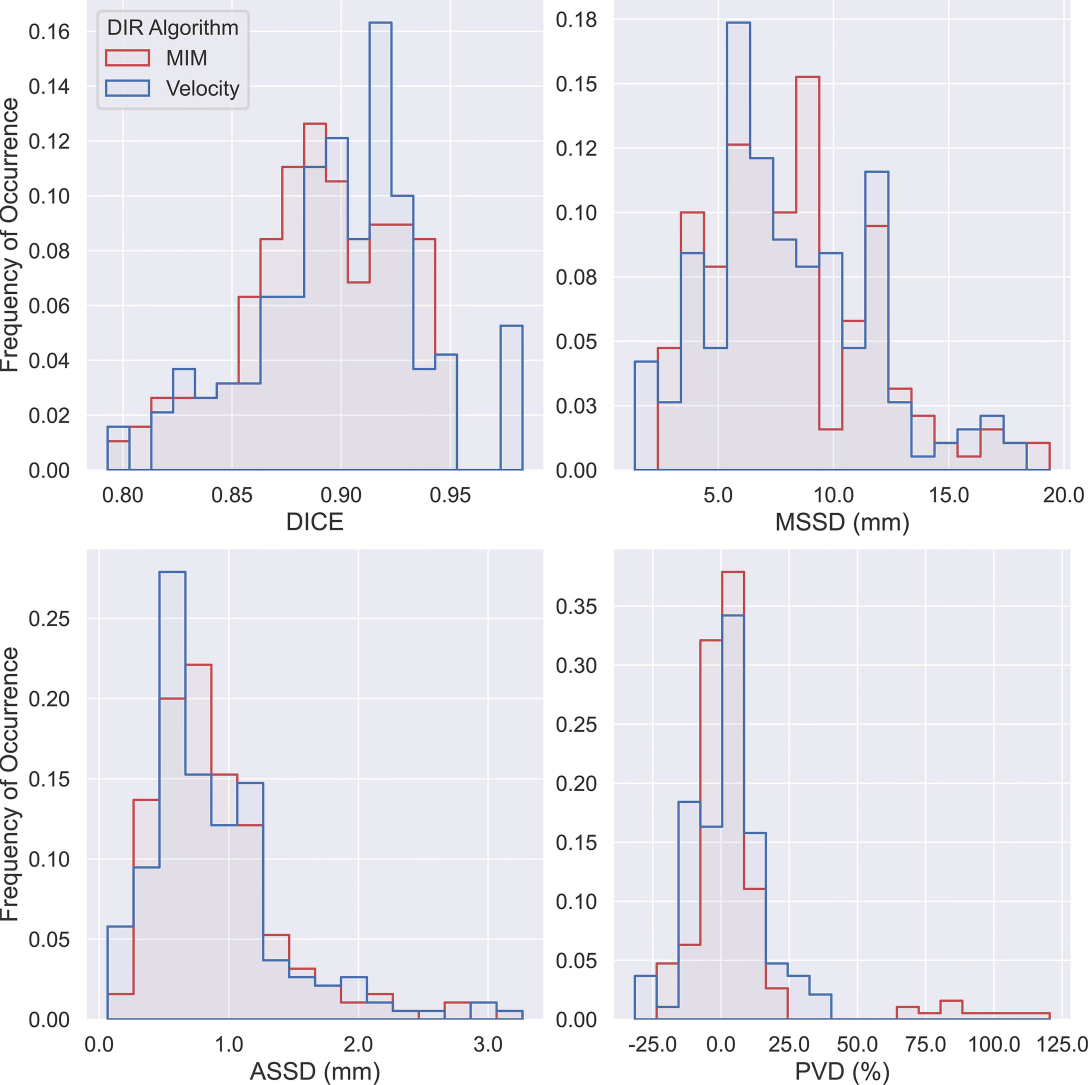
Histogram of Dice coefficient (DICE), maximum symmetric surface distance (MSSD), average symmetric surface distance (ASSD), and percent volume difference (PVD) from pairwise comparisons of all 190 auto-propagated internal gross tumor volumes (AP-IGTVs) relative to their corresponding ground-truth internal gross tumor volumes (GT-IGTVs) for the two employed deformable image registration (DIR) algorithms (MIM in red; Velocity in blue).

A summary flow chart of the study process can be found in **Figure 1**. LA-NSCLC lesions of the study patients exhibited a wide variation in size, shape, and anatomical location. Patient characteristics are summarized in **Table 1**. GT-IGTV volumes ranged from 9.2 to 316.0 cm^3^ with a median of 77.2 cm^3^, while ranging from 8.3 to 436.6 cm^3^ with a median of 78.0 and from 8.4 to 429.8 cm^3^ with a median of 76.3 cm^3^ for AP-IGTV volumes generated by the MIM and velocity DIR algorithms, respectively. AP-IGTVs derived from the different respiratory phases using the two DIR algorithms in comparison to the GT-IGTV for one lesion representative of the study cohort are illustrated in **Figure 2**. The overall variations of the generated AP-IGTVs from their corresponding GT-IGTVs for both DIR algorithms are reflected in the distribution of the accuracy measures as shown in **Figure 3**. As illustrated, the vast majority (>85%) of auto-propagated tumor volumes for either algorithm achieved DICE above 0.850 and ASSD less than 1.5 mm. This demonstrates that auto-propagated target volumes from both algorithms attained substantial spatial overlaps with their respective ground-truth volumes while approximating their respective ground-truth surface with millimeter to submillimeter accuracy on average. Nonetheless, for both DIR algorithms, over a quarter of the auto-propagated volumes were associated with MSSD greater than 1 cm and PVD beyond an absolute value of 10%, indicating the risk of DIR-based auto-propagation in under- and/or overestimating the ground-truth IGTVs.

**Table 1:** Patient characteristics (n = 19).

Demographic		Occurrence	Percentage
Sex	Male	11	57.9%
	Female	8	42.1%
TNM stage	IIIA	10	52.6%
	IIIB	9	47.4%
T category	T0	1	5.3%
	T1	1	5.3%
	T2	5	26.2%
	T3	6	31.6%
	T4	6	31.6%
N category	N1	2	10.5%
	N2	11	57.9%
	N3	6	31.6%
Tumor location	Central versus peripheral		
	Central	14	73.7%
	Peripheral	5	26.3%
	Superior versus inferior		
	Superior	9	47.4%
	Inferior	10	52.6%

**Figure 4:**
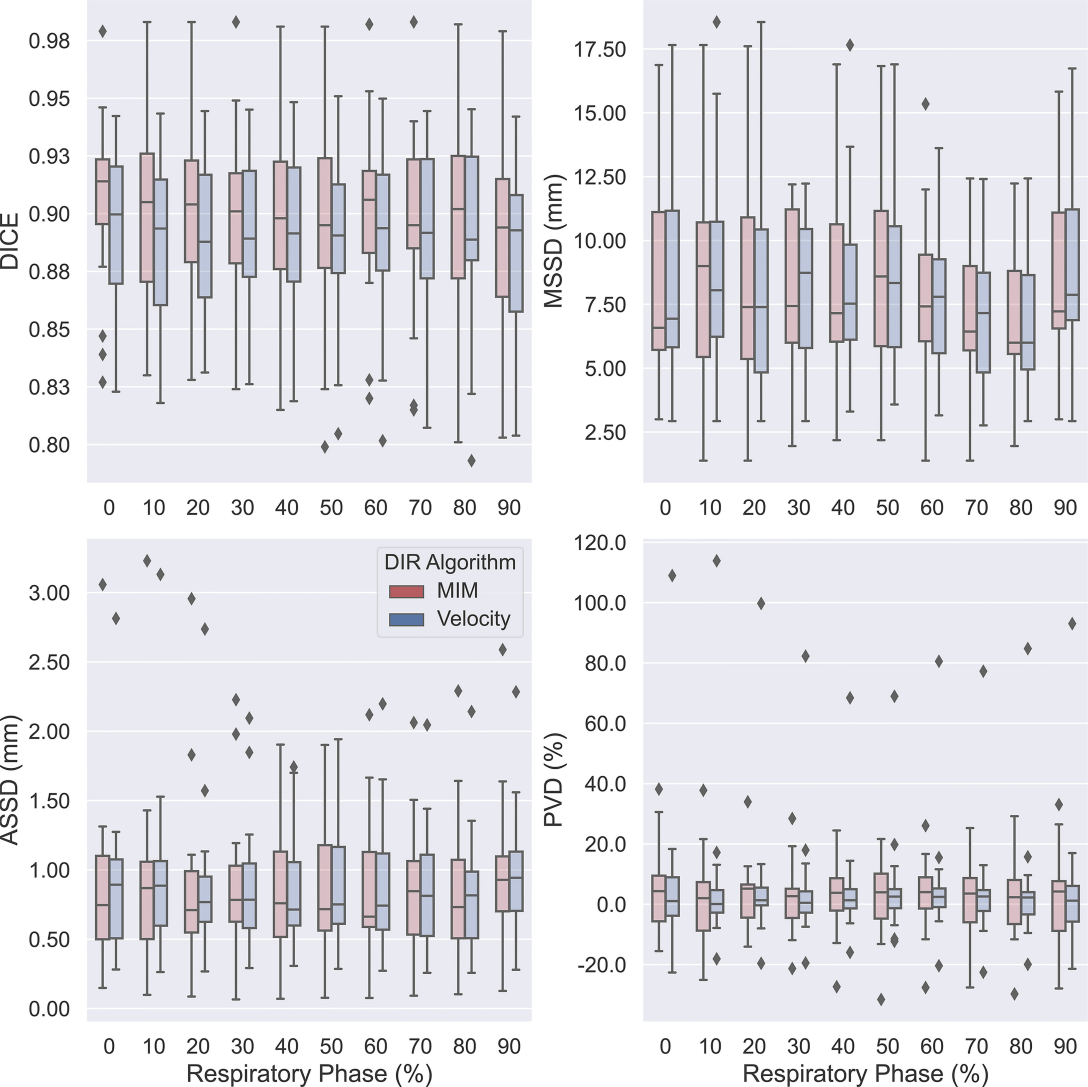
Boxplots comparing accuracies of auto-propagated internal gross tumor volumes (AP-IGTVs) from the two employed deformable image registration (DIR) algorithms—MIM (red) and Velocity (blue)—relative to their corresponding ground-truth internal gross tumor volumes (GT-IGTVs) in terms of Dice coefficient (DICE), maximum symmetric surface distance (MSSD), average symmetric surface distance (ASSD), and percent volume difference (PVD) between different respiratory phases for the study cohort. On each box, the central mark indicates the median, and the top and bottom edges of the box indicate the 25th and 75th percentiles, respectively.

**Figure 5:**
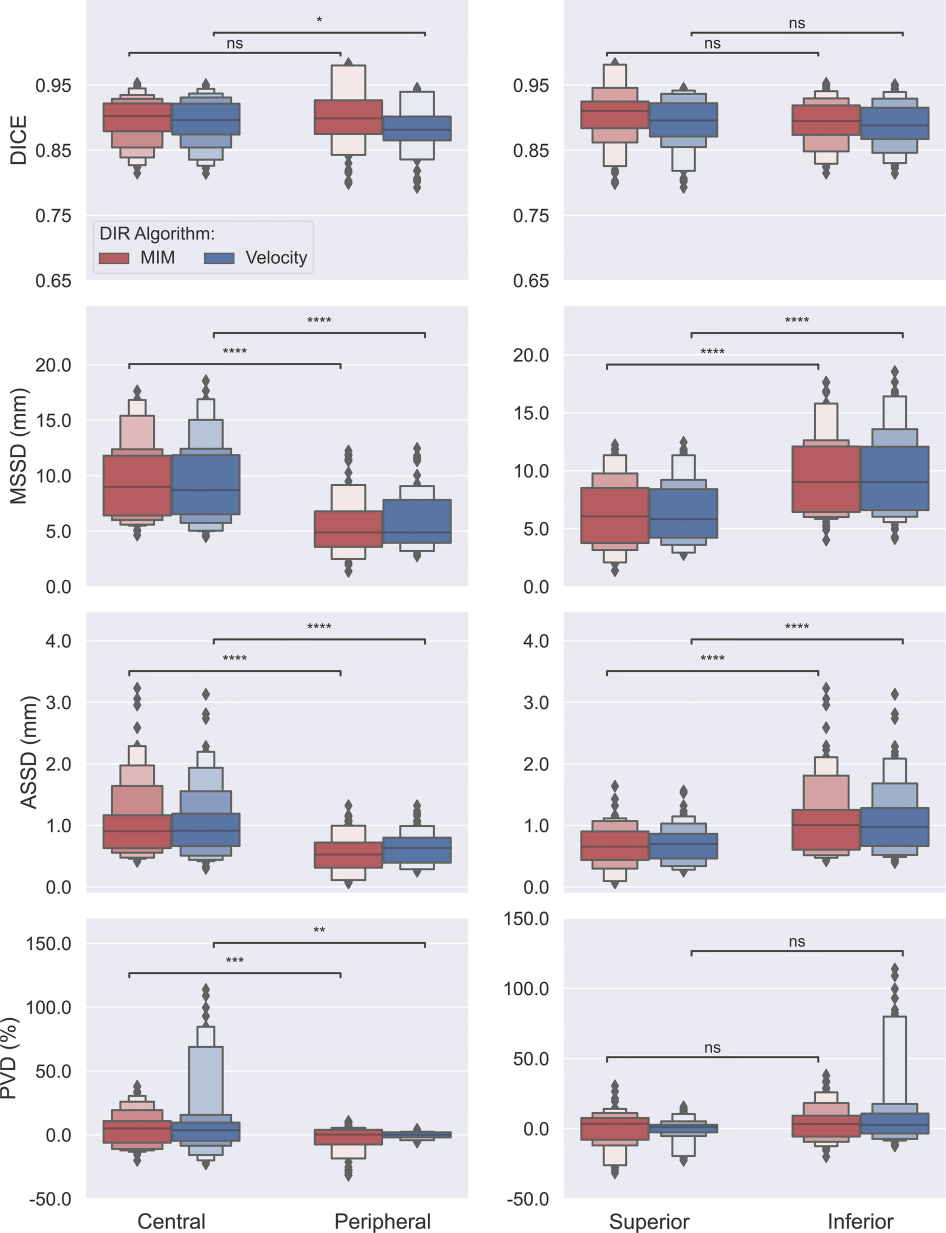
Box plots comparing accuracies of auto-propagated internal gross tumor volumes (AP-IGTVs) from the two employed deformable image registration (DIR) algorithms—MIM (red) and Velocity (blue)—relative to their corresponding ground-truth internal gross tumor volumes (GT-IGTVs) in terms of Dice coefficient (DICE), maximum symmetric surface distance (MSSD), average symmetric surface distance (ASSD), and percent volume difference (PVD) along the central–peripheral, and superior–inferior dimensions. On each box, the central mark indicates the median, and the top and bottom edges of the box indicate the 25th and 75th percentiles, respectively. For each box pair comparison, NS: not significant at level of 0.05; *: *p* < 0.05; **: *p* < 0.01; ***: *p* < 0.001; ****: *p* < 0.0001.

The obtained data on measures of accuracy between AP-IGTVs and their corresponding GT-IGTVs were also pooled according to respiratory phase and anatomical tumor location to examine whether there existed any temporal or spatial dependences associated with DIR-based auto-propagation of IGTVs. **Figure 4** shows boxplots comparing the employed accuracy metrics, including DICE, MSSD, ASSD, and PVD, between the 10 different respiratory phases for the two DIR algorithms. As mentioned previously, the 10 phases represent 10 equally spaced time points over the course of a full respiratory cycle, with 0% being peak inhalation and 50% end of exhalation. The effect of the respiratory phase on the accuracy of DIR auto-propagated IGTVs manifested low to moderate fluctuations in both algorithms. With the MIM DIR algorithm, the mean of DICE among the 10 respiratory phases ranged from 0.892 to 0.905, mean of MSSD from 7.2 to 8.7 mm, mean of ASSD from 0.8 to 1.0 mm, and mean of absolute PVD from 8.4% to 12.3%. As to the velocity DIR algorithm, the mean of DICE among the 10 respiratory phases ranged from 0.885 to 0.894, mean of MSSD from 7.2 to 9.0 mm, mean of ASSD from 0.9 to 1.0 mm, and mean of absolute PVD from 8.1% to 12.9%. Moreover, the Kruskal–Wallis test showed no significant differences in DICE, MSSD, ASSD, and PVD between the 10 respiratory phases for both DIR algorithms with all *ps* > 0.9000. **Figure 5** presents boxplots comparing the accuracy metrics employed between the anatomical locations where the primary tumoral masses were situated. First, the DICE between AP-IGTVs and GT-IGTVs did not show significant differences along any of the anatomical dimensions examined except for a difference in central and peripheral tumors with AP-IGTVs created by the Velocity algorithm, indicating that the anatomical location of the tumor may have limited influence on DIR-based auto-propagation in generating IGTV accurate at a large geographic scale. Second, MSSD and ASSD both demonstrated significantly higher values with centrally located primary tumors compared to peripheral tumors (*ps* < 0.0001). Likewise, the inferiorly located tumors had higher MSSD and ASSD values compared to superior tumors (*ps* < 0.0001). The MSSD and ASSD findings were consistent in both DIR algorithms. With MSSD and ASSD emphasizing differences in surface morphology between AP-IGTVs and their corresponding GT-IGTVs, this implies that on geographically small scales there might exist a certain degree of spatial dependence in DIR-based auto-propagation of the GTV. Furthermore, it was also observed that the centrally located tumors differed significantly from the peripherally located ones with a significantly higher PVD (*p* < 0.001). Coupling this with the results regarding MSSD and ASSD, this may suggest that caution should be exerted in using DIR-based auto-propagation to generate an IGTV for centrally located LA-NSCLC tumors.

**Figure 6:**
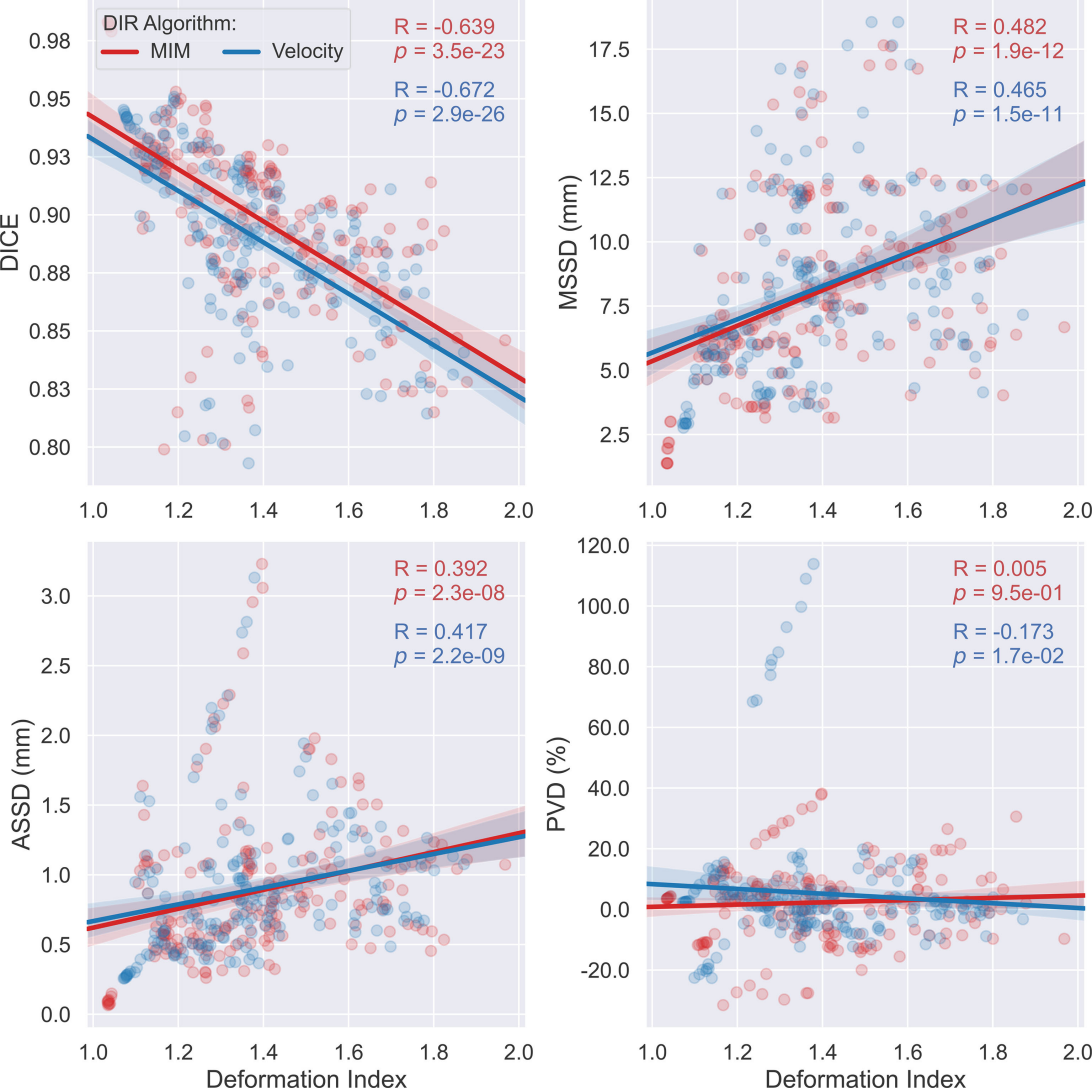
Scatter plots with linear regression (95% confidence intervals of the predicted mean) outlining the distribution of Dice coefficient (DICE), maximum symmetric surface distance (MSSD), average symmetric surface distance (ASSD), and percent volume difference (PVD) versus the deformation index for the two employed deformable image registration (DIR) algorithms—MIM (red) and Velocity (blue). Each dot corresponds to a different single respiratory phase for one individual patient.

Scatter plots with superimposed linear regression showing the trend of correlations between the studied accuracy metrics and the deformation index are illustrated in **Figure 6**. As depicted, for both algorithms, DICE drops while MSSD and ASSD rise with respect to the increasing deformation index. Further assessment on the statistical strength of the associations between these accuracy metrics and the deformation index using the Spearman’s rank correlation test demonstrated that three of the four examined metrics, namely, DICE, MSSD, and ASSD, significantly correlate with the deformation index with Spearman’s correlation coefficient (R) of -0.639, 0.482, and 0.392 for the MIM DIR algorithm and -0.672, 0.465, and 0.417 for the Velocity DIR algorithm (all *ps* < 0.0001). These results substantiate the notion that the accuracy of auto-propagated IGTV deteriorates as the amount of deformation required to register the tumor between different respiratory phases increases.

**Figure 7:**
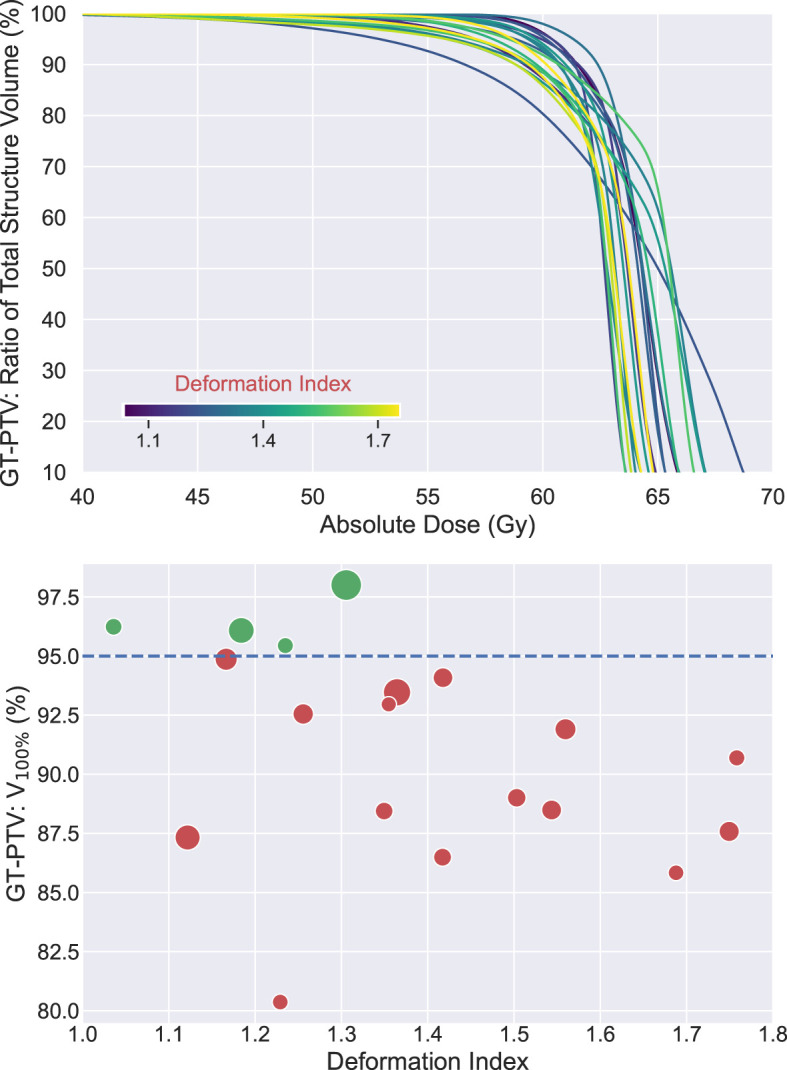
Top: dose volume histograms (n = 19) of ground-truth planning target volumes (GT-PTVs) created with volumetric modulated arc therapy (VMAT) plan targeting corresponding auto-propagated planning tumor volume (AP-PTV). All plans met dose prescription of V_100%_ ≥ 95% of AP-IGTV and nearby organ at risk (OAR) constraints. Each line colored according to auto-propagated internal gross target volume (AP-IGTV) deformation index. Bottom: scatter plot of V_100%_ values of GT-PTVs as a function of deformation index of the corresponding AP-IGTV. Dots in green above the blue dashed line met planning objective of V_100%_ ≥ 95%, whereas dots in red below the blue dashed line failed to meet the constraint. Size of the dots is proportional to the size of ground-truth internal gross target volume (GT-IGTV).

To evaluate the dosimetric consequences of the erring patterns described, VMAT plans were made, as mentioned above, to target the AP-PTV derived from the AP-IGTV that had DI closest to the overall median DI among all the AP-IGTVs created by both DIR algorithms for each patient. All VMAT plans created met the RTOG 0617 prescription constraints to the targets and nearby OARs (3). DVHs of the 19 GT-PTVs are shown in the top panel of **Figure 7**, while in the bottom shows a scatter plot displaying V_100%_ of the GT-PTVs as a function of deformation index of the corresponding AP-IGTVs used for planning. Although all the VMAT plans satisfied the prescription constraints of V_100%_ ≥ 95% for the AP-PTVs, GT-PTVs in only 4 out of the 19 (21%) patients met this constraint, namely, that 79% of the GT-PTVs (dots labeled in red below the blue dashed line) failed to meet the prescription constraint.

## Discussion

Outcomes following definitive chemoradiation therapy for LA-NSCLC are poor with a median progression-free survival under 1 year highlighting the urgent need for treatment advances for this disease (3). Immunotherapy will play a role, but local failures occur in 30%–40% of LA-NSCLC patients receiving definitive chemoradiation suggesting that improvements in local treatment with RT are necessary to further improve outcomes (3, 6). A 4DCT image can be used to generate an ITV that accounts for lung tumor movement due to respiratory motion enhancing radiation targeting (11). In clinical practice, auto-propagation algorithms are often used to produce an ITV from a manual contour on a single RP 3DCT image. Auto-propagation algorithms are efficient and accurate in early-stage NSCLC (20), but they have not been studied in a homogeneous pool of LA-NSCLC patients nor been analyzed for tumor spatiotemporal characteristics and dosimetric consequences.

In this study, the 4DCTs of 19 patients with LA-NSCLC treated with definitive chemoradiation were employed for this purpose. The VoxAlign Deformation Engine from MIM Maestro^®^ v7.1.3 (MIM Software, Cleveland, OH) and the elastic B-spline deformation algorithm from Velocity^™^ oncology imaging informatics system v4.1 (Varian Inc., Palo Alto, CA), two of the most frequently applied commercially available DIR-based IGTV auto-propagation algorithms, were adopted to generate IGTVs using manual GTV contours from each RP of all 19 individual patients. Spatial incoherencies between the resultant AP-IGTVs and their corresponding GT-IGTVs as measured with respect to spatial overlap (DICE), surface deviation (MSSD and ASSD), and volume difference (PVD) for each of the acquired 3DCT scans were pooled by respiratory phase, anatomic tumor location, and deformation index, with each being examined for whether and to what extent it impacts IGTV propagation accuracy. One important finding gleaned from the present study was the observation that DICE, MSSD, and ASSD are all significantly correlated with the deformation index with both the MIM DIR algorithm (Spearman’s rank correlation coefficient: -0.639, 0.482, 0.392, respectively; all *ps* < 0.0001) and the Velocity DIR algorithm (Spearman’s rank correlation coefficient: -0.672, 0.465, 0.417, respectively; all *ps* < 0.0001). These highly statistically significant relationships, especially the one between MSSD and the deformation index, bear important clinical implications. MSSD is of particular importance for target volume design as it determines the maximum margin of error by selecting the largest of all calculated distances between the surfaces of one AP-IGTV and its corresponding GT-IGTV. This finding, therefore, implies that AP-IGTVs with a high deformation index ensuing from the great motion envelope of their pertaining GTVs may need larger safety margins or closer physician review to avoid RT target miss or overdosing normal tissues. In other words, the deformation index may have the potential to be a predictor of accuracy for DIR-based IGTV auto-propagation and thereby could be used to personalize the internal margin design for planning radiotherapy in LA-NSCLC. This observation of the current study also corresponds to previous investigations (38, 39), which reported that tumor motion complicates ITV generation, often resulting in increased delineation variability and exacerbated dosimetric uncertainty in RT of NSCLC, irrespectively of the motion management techniques being employed.

In addition to the deformation index, RP and anatomic tumor location were assessed for their impacts on the seen differences between the AP-IGTVs and their corresponding GT-IGTVs. Notably, it was found that the RP used to initialize the auto-propagation does not seem to have any statistically significant influences on the analyzed differences between the AP-IGTVs and GT-IGTVs, even though previous studies have suggested the importance of the choice of end-expiration RP (17, 40) or end-inspiration RP (41) for IGTV auto-propagation on the robustness and accuracy of formulating the target volume in free-breathing RT of NSCLC. With the auto-propagation algorithm used herein, these results demonstrate that any RP, at least for the LA-NSCLC cohort studied, can be used for initial manual GTV contours with no significant difference in the accuracy of IGTV produced.

On the other hand, anatomical tumor location was found to have a certain degree of impact on the observed differences between the AP-IGTVs and GT-IGTVs. MSSD, ASSD, and PVD were found to be significantly lower for peripheral tumors compared to central tumors. Similarly, MSSD and ASSD were significantly lower for superior tumors compared to inferior tumors. These findings could be explained by the increase in tumor motion in the lower lobes, primarily in the superior–inferior direction (8, 42–44). The structural location within the ITV accounting for differences between AP-IGTV and GT-IGTV was not evaluated in this study. Other analyses have demonstrated that the superior and inferior ends of the ITV structure have the most discrepancy with auto-propagation (40, 45). Both the primary tumor and gross nodal disease were analyzed in this study, but nodal IGTVs may have larger differences between auto-propagated IGTVs and GT-IGTVs (17). It is also possible that other factors that influence tumor motion, such as patient age and gender (7), are associated with differences between GT-IGTVs and AP-IGTVs.

Errors in auto-propagating the IGTV may lead to further errors downstream in the treatment planning and delivery process. The dosimetric implications of this discrepancy between AP-IGTVs and GT-IGTVs was demonstrated in the present study with 79% of VMAT plans created by targeting the AP-PTV which did not cover the corresponding GT-PTV with V_100%_ ≥ 95%. The clinical importance of this dosimetric discrepancy should be explored further. For example, the deformation index appears to predict not only error in AP-IGTV generation but also dosimetric underdosage of the GT-PTV. A larger sample size than the present study is necessary to make a conclusion of this latter point, although there appears to be a positive trend between deformation index and GT-PTV underdosage. It has been shown that planning with 4DCT can minimize the risk of under-radiated tumor volumes, but optimizing ITV auto-propagation may further reduce this risk (46). It is difficult to predict local failure in LA-NSCLC, but approximately 38% of locoregional failures are marginal occurring outside the ITV within 1 cm of the PTV (47). Further study is necessary to determine if these failures could be prevented with more accurate target volumes. Accurate ITV propagation is particularly important for patients treated with proton beam radiation therapy as the dosimetry is less forgiving to tumor motion, although this has not been shown to be of clinical consequence (47). In addition to minimizing the risk of marginal failures, optimizing dosimetric parameters can lead to decreased rates of esophagitis (48) and radiation pneumonitis (49).

Limitations of the present study include the evaluation of only two, although generally accepted and widely used, commercially available DIR algorithms for DIR-based auto-propagation, and thus the results may not be entirely applicable to other IGTV propagation algorithms. Because all tumors were LA-NSCLC in the present study, spatial and temporal influences described cannot be directly generalized to early-stage or other histologies. For example, Maximum intensity projections (MIP) has been shown to be less reliable in generating ITVs for more advanced tumors compared to early-stage tumors (50). Furthermore, the ITVs analyzed in this study were created directly from GTV respiratory motion as described elsewhere (17, 41) rather than creating an ITV from a CTV volume. Finally, the presented results should be interpreted with prudence given that the study cohort was composed of patients from a single institution, which might be subject to potential selection biases. Nonetheless, the findings of this study are strengthened by the inclusion of a farily large number of patients with homogeneous disease stage and histology.

## Conclusions

For patients with LA-NSCLC, errors in DIR-based IGTV auto-propagation are present to varying degrees and exhibit dependencies on the deformation index and intrathoracic tumor location. Interestingly, the respiratory phase used to initialize AP-IGTV propagation was found not to correlate significantly with analyzed differences with the GT-IGTV. These findings may allow for the formation of new hypotheses toward achieving robust and more accurate RT targeting in LA-NSCLC.

## Data Availability Statement

The raw data supporting the conclusions of this article will be made available by the authors, without undue reservation.

## Ethics Statement

Ethical review and approval was not required for the study on human participants in accordance with the local legislation and institutional requirements. Written informed consent for participation was not required for this study in accordance with the national legislation and the institutional requirements.

## Author Contributions

All authors contributed to the study’s conception and design. Material preparation, data collection, and analysis were performed by BR, BS, and FY. The first draft of the manuscript was written by BR and FY. All authors commented on previous versions of the manuscript. All authors reviewed and approved the final manuscript.

## Conflict of Interest

The authors declare that the research was conducted in the absence of any commercial or financial relationships that could be construed as a potential conflict of interest.

## Publisher’s Note

All claims expressed in this article are solely those of the authors and do not necessarily represent those of their affiliated organizations, or those of the publisher, the editors and the reviewers. Any product that may be evaluated in this article, or claim that may be made by its manufacturer, is not guaranteed or endorsed by the publisher.
